# Phage–Antibiotic Combination Therapy against Recurrent *Pseudomonas* Septicaemia in a Patient with an Arterial Stent

**DOI:** 10.3390/antibiotics13100916

**Published:** 2024-09-24

**Authors:** Ulla Elina Otava, Laura Tervo, Riikka Havela, Liisa Vuotari, Matti Ylänne, Annette Asplund, Sheetal Patpatia, Saija Kiljunen

**Affiliations:** 1 Department of Internal Medicine, Tampere University Hospital, 33520 Tampere, Finlandlaura.tervo@pirha.fi (L.T.); riikka.havela@tuni.fi (R.H.); 2 Faculty of Medicine and Health Technology, Tampere University, 33520 Tampere, Finland; liisa.vuotari@pirha.fi; 3 Department of Clinical Physiology and Nuclear Medicine, Tampere University Hospital, 33520 Tampere, Finland; 4 Human Microbiome Research Program, Research Programs Unit and Medicum, Faculty of Medicine, University of Helsinki, 00014 Helsinki, Finlandsheetal.patpatia@helsinki.fi (S.P.); 5 PrecisionPhage Ltd., 40500 Jyväskylä, Finland

**Keywords:** arterial stent, neutropenia, meropenem, phage therapy, *Pseudomonas aeruginosa*, septicemia

## Abstract

**Background:** Intravascular stent infections are often associated with high risks of morbidity and mortality. We report here a case of a patient with an arterial stent and recurrent *Pseudomonas* septicaemias successfully treated with phage–meropenem combination therapy. **Methods:** A 75-year-old female with arteriosclerosis and comorbidities went through a femoropopliteal bypass with prosthesis in the right inguinal area. After the bypass, she developed a recurring *Pseudomonas aeruginosa* infection and also neutropenia during different antibiotics. A rapidly growing pseudoaneurysm in the right inguinal area led to an emergency intra-arterial stent placement during blood stream infection, later suspected to host a *P. aeruginosa* biofilm. Removing the stent was deemed precarious, and phage therapy was considered as a compassionate treatment option. A three-phage cocktail infecting the *P. aeruginosa* strain was prepared and administered intravenously together with meropenem for two weeks, after which, a ten-month follow-up was carried out. **Results:** No adverse reactions occurred during the phage therapy treatment, while infection markers were normalized. In addition, recovery was seen in a PET-CT scan. During the 10-month follow-up, no further *P. aeruginosa* septicaemias occurred. **Conclusions:** Phage–meropenem combination therapy was thus found safe and effective in the treatment of recurrent *Pseudomonas* septicaemia in a patient with an arterial stent.

## 1. Introduction

Intravascular stent and graft infections are severe infectious diseases associated with high morbidity and mortality [1]. The frequency of graft infections depends on the anatomic location of the graft, but the usual infection rate is from 1% to 6% [2]. The most common causative agents in these infections are coagulase-negative staphylococci, *Staphylococcus aureus*, and Gram-negative bacilli, out of which *Pseudomonas aeruginosa* is the most common [2].

*P. aeruginosa* is an opportunistic pathogen often associated with hospital-acquired infections, pulmonary infections of immunocompromised patients, and bloodstream infections [3]. *P. aeruginosa* is a member of the so-called ESKAPE group of pathogens, for which antibiotic resistance makes the treatment of infections especially challenging [4]. In 2019, *P. aeruginosa* was found to be one of the six leading pathogens responsible for antimicrobial resistance -associated deaths [5]. *P. aeruginosa* is recognized for its ability to form biofilms, which makes it even more difficult to eradicate [6].

As the treatment of infections caused by *P. aeruginosa* with antibiotics is becoming more and more challenging, other alternatives need to be studied. Phage therapy, the treatment of bacterial infections with viruses that infect and lyse bacteria (phages or bacteriophages), was already discovered over 100 years ago. The application of phage therapy in Western medicine ceased after the commercialization of antibiotics, but it has experienced a renaissance upon the increased antibiotic resistance of pathogenic bacteria [7]. *P. aeruginosa* has rapidly become one of the most often recognized target bacteria for phage therapy research. In the report by Pirnay et al. (2024), describing the outcomes of 100 phage therapy cases, *P. aeruginosa* was involved in 49 out of the 100 cases [8]. However, the results of phage treatments have varied from case to case, and the success rates of phage therapy in general are estimated to be approx. 50–80% [8,9,10]. Reasons for failure include the emergence of phage resistance, the neutralization of phages by the immune system, and an increase in biofilm production [8,11]. As studies reporting phage treatments of *P. aeruginosa* still remain controversial, more thorough understanding of phage therapy of *P. aeruginosa* infections is still needed.

Here, we present a case report of a Finnish 75-year-old female suffering from recurrent *Pseudomonas* septicaemia, presumably resulting from a biofilm in the arterial stent. The patient was successfully treated with phage–meropenem combination therapy, and the success was confirmed with PET-CT imaging. No obvious side effects were detected during the treatment.

## 2. Case Report

### 2.1. The Patient

The patient was a 75-year-old woman suffering from diabetes mellitus type 2 combined with neuropathy and universal arteriosclerosis caused by 20 pack-years of cigarette smoking earlier in her life. She had a non-healing ischemic wound on her right heel. She had undergone colectomy a few years earlier due to an ischemic attack, and a femoropopliteal bypass surgery with artificial material was performed in her left lower limb seven years prior to the recent events. The patient had a physiological pacemaker due to dilated cardiomyopathy and she was allergic to the antibiotics amoxicillin, clindamycin, and metronidazole.

**Figure 1:**
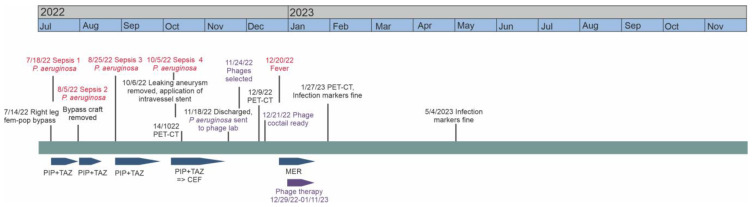
Timeline of the patient’s case. Episodes of sepsis or fever are highlighted in red, and the courses of antibiotics and phage therapy are represented by blue and purple arrows, respectively. PIP: piperacillin; TAZ: tazobactam; CEF: ceftazidime; MER: meropenem.

In summer 2022, the patient had a non-healing ischemic wound on her right heel. Femoropopliteal bypass with prosthesis was performed in the right inguinal area. Three days later, she had her first septicemia caused by *P. aeruginosa* (see timeline in Figure 1). The pathogen was susceptible to all tested antimicrobial agents except trimetoprim + sulpha. The patient received a two-week standard treatment with intravenous piperacillin (4 g at 8 h intervals, i.e., 4 g q8h) iv), combined with tazobactam (0.5 g q8h iv). Infection markers were normalized during the treatment but elevated again some days after the end of the course. *P. aeruginosa* was found in her bloodstream again. The newly applied arterial prosthesis was removed. *P. aeruginosa* of an otherwise similar susceptibility pattern, but resistant to levofloxacin, was growing in the samples taken from the inguinal area during the operation. Therefore, intravenous piperacillin–tazobactam was given (4 g/0.5 g q8h iv) for 10 days.

Ten days after the last dose of the antibiotics, the patient had a high fever, and *P. aeruginosa* was found again in the bloodstream. Cardiac ultrasound was performed but no signs of intracardiac infection or vegetation in the pacemaker wire were found. A whole-body PET-CT was performed, showing isotope activity in the right inguinal area, right sacroiliac (SI) joint, and near the unhealed wounds of the right limb, indicating ongoing infection No activity was detected near the pacemaker or the arterial prosthesis of the left calf. A four-week treatment with piperacillin–tazobactam was given. The wound in the right inguinal area was treated with VAC (vacuum-assisted closure) treatment, and it was starting to heal.

After a ten-day pause in antibiotics, *P. aeruginosa* was found again in the blood of the patient, and higher-dose piperacillin–tazobactam (4 g/2 g q6h iv) was started. Simultaneously, a pseudoaneurysm started to grow rapidly near the still-open inguinal surgical wound at the removal site of the former right arterial prosthesis. The aneurysm, suspected to be linked to local infection, was surgically repaired with an intra-arterial stent. *P. aeruginosa* was growing in the samples taken during the operation near the pseudoaneurysm.

During this course of piperacillin–tazobactam, a significant neutropenia started to develop. Neutrophil growth factor filgrastim was given, but the benefit lasted for only a few days. The neutropenia was suspected to be antibody-mediated, induced by the penicillin group antibiotic piperacillin. The treatment was changed to ceftazidime (2 g q8h iv), but neutropenia reappeared. Even though meropenem was still an option, other treatment alternatives began to be considered.

**Figure 2:**
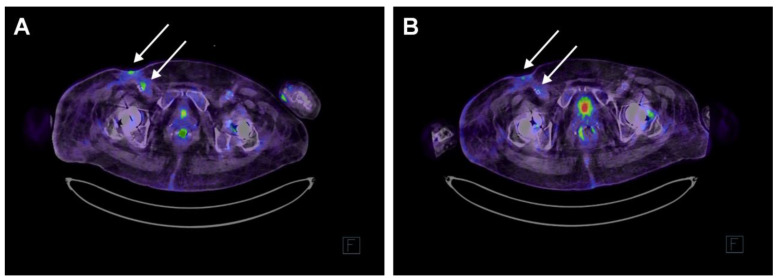
Axial fused PET-CT image at level of inguinal region. (**A**) Two weeks prior to the phage treatment. In the right groin, next to the a. iliaca externa stent, there was minor FDG (fluorodeoxyglucose) uptake SUVmax (maximum standardized uptake value) 4.9. On the surface of the skin of the right groin, there was a slightly higher uptake SUVmax 8.1. (**B**) PET-CT scan two weeks after the phage therapy course. In the area of the vascular stent in the right groin/femoral region, there was no residual activity to be mentioned. In the right groin, there was minor uptake superficially in the skin/scar area SUVmax 3.8. Arrows pinpoint the locations with altered metabolic activities. As the marker of glucose metabolism, 18-FDG 244 MBq was used, and imaging was performed with Siemens Biograph Vision.

Because the arterial stent was placed during the ongoing intravenous infection by *P. aeruginosa*, we suspected that the stent hosted *P. aeruginosa* bacteria, possibly a biofilm, thus causing the persistent infection. Removing the stent was considered to cause an unacceptably high risk of severe bleeding. Therefore, phage therapy was considered as a compassionate treatment option. At this point, the patient was stable and was discharged from the hospital. The surgical wound in the right inguinal area was still open, though getting smaller. The right lower limb also had two other superficial ischemic wounds. The right SI joint and lower back were still painful. At that time, a new whole-body PET-CT (Figure 2A) was performed, proving ongoing infection activity near the right inguinal area linked to the stent.

The next fever episode began four weeks later, and meropenem (1 g q8h iv) was administered by the Home Hospital Unit. Unfortunately, this also caused neutropenia that was not resolved with filgrastim, though not as severe as that with piperacillin.

### 2.2. Phage Cocktail HFC-Pae10

Phages fNenPA2p2 and fNenPA2p4 and fGstaPae02 were selected to form the HFC-Pae10 phage cocktail based on their clear plaque formation in the patient isolate #7250. fNenPA2p2 and fNenPA2p4 have 66.1 kb and 66.2 kb genomes, respectively, and they both belong to the genus *Pbunavirus*. fGstaPae02 has a genome of 93.1 kb and it belongs to the genus *Pakpunavirus*. None of the phage genomes carry genes related to bacterial virulence, antibiotic resistance, or phage lysogenic life cycle. The total titer of the cocktail in strain #7250 was 10^7^ PFU/mL, and the endotoxin concentration was 6350 EU/mL.

**Figure 3:**
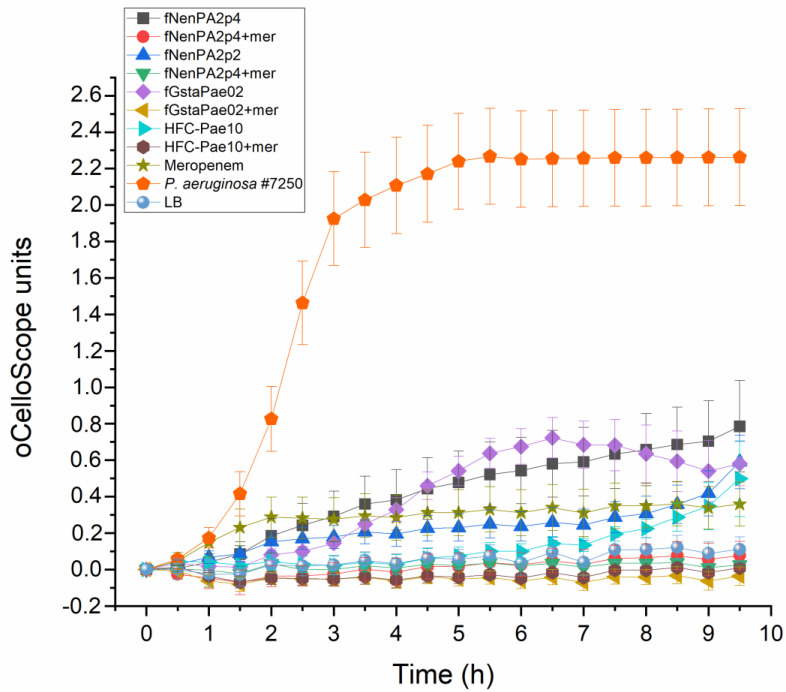
Infectivity of phages fNenPAp2, fNenPAp4, and fGstaPae02, and the phage cocktail HFC-Pae10 in *P. aeruginosa* strain #7250. Phage infection is measured with individual phages and the cocktail in LB medium with and without meropenem (200 µg/mL). Each sample was analyzed in triplicate, and mean and standard deviation are shown. Calculations and visualization were performed with OriginPro 2022b.

The potency of the HFC-Pae10 cocktail and the three phages individually with regard to infecting *P. aeruginosa* #7250 in the presence of meropenem (200 µg/mL) was verified in a liquid culture assay. The combination of phages and meropenem prevented the bacterial growth more efficiently than phages or the antibiotic alone, indicating a clear synergetic effect (Figure 3).

### 2.3. Phage Treatment of the Patient

After a week of meropenem treatment (Figure 1), administration of the phage product was started (Day 0 in Table S1). The phage treatment was given once a day intravenously. The daily phage dose was 7.5 × 10^6^ PFU, corresponding to a total of 4763 EU endotoxin. No immediate adverse effects were observed. Meropenem (1 g q8h iv) was continued alongside the phages. The first two doses of the phage were given in an inpatient setting. Because the patient had no adverse reactions, the intravenous phage and antibiotic treatment was continued by the Home Hospital Unit. The treatment and follow-up were performed without incident.

After the two-week phage–meropenem course, the patient did not have a fever. She was in better health and moving outdoors. In a whole-body PET-CT two weeks after the beginning of the phage treatment, the activity in the inguinal area was markedly decreased (Figure 2B). In an outpatient clinic call one month after the end of treatment, she was in full recovery of symptoms.

Serum samples taken prior to the first phage infusion and five weeks after the end of the phage therapy course were tested for the presence of phage-neutralizing antibodies. Serum samples taken before and after the phage treatment caused titer drops of 54% and 68%, respectively, indicating that no major phage-neutralizing effect was formed.

Five months after the therapy, an inguinal ultrasound was performed. No signs of infection relapse were found, and the infection markers were within normal limits. During a 10-month follow-up, the inguinal arterial stent previously suspected to host a biofilm causing recurring *P. aeruginosa* septicaemias remained in place without any noticeable issues.

## 3. Discussion

The incidence of bacterial infections associated with cardiac and vascular surgery is usually low, below 6%, but the mortality rates in these infections may be as high as 75% [2,12]. The management of these infections may require both antimicrobial and surgical therapy. However, surgical therapy can cause a high risk for compromised patients; therefore, it is not always a feasible option [2,12]. If antimicrobial therapy for these patients fails due to, e.g., antimicrobial resistance of the pathogen or severe side effects on the patient, the treatment options may be limited.

Phage therapy has recently been studied as a potential treatment in vascular graft- and/or stent-associated infections [13,14]. As clinical studies on the topic are lacking, and conducting such clinical studies may be extremely challenging, most data regarding the suitability of phage therapy for infections associated with cardiac and vascular surgery come from individual case studies. Rubalskii et al. (2020) presented a phage therapy case series of eight patients with implant- and transplant-associated infections. Causative infectious agents in the study were *P. aeruginosa*, *Staphylococcus aureus*, *Enterococcus faecium*, *Klebsiella pneumoniae*, and *Escherichia coli*. Each of these eight patients was treated with phage–antibiotic combination therapy, and five of the patients completely recovered from the infection. The three patients who did not recover died later due to either transplant failure or bacterial infections, but it was not certain whether the infections were caused by the same strains that caused the original infections [15]. Grambow et al. (2022) published the successful phage therapy treatment of an *S. aureus*-infected stent graft in the thoracic aorta [16], while Blasco et al. (2023) reported phage therapy failure in a patient with a prosthetic vascular graft infection caused by *P. aeruginosa* [11]. These examples illustrate the need for more studies on vascular surgery -associated phage therapy in order to gain better understanding of successful application procedures and limitations that may result in treatment failures.

In this case study, we present a phage–antibiotic combination treatment of a patient with an inguinal intra-arterial stent infected with *P. aeruginosa* that resulted in several episodes of sepsis despite antibiotic treatments. During the two-week phage therapy course, no adverse reactions took place, the infection markers were normalized, and the patient did not have further episodes of septicemia. *P. aeruginosa* was eradicated from the non-removable right inguinal femoral artery stent. No neutralization of the phage cocktail by the immune system was observed after the treatment despite the iv application of the phage, even though the emergence of phage-neutralizing antibodies has been observed in other studies [17,18]. Phage–antibiotic combinations have earlier been shown to be more efficient in bacterial killing and biofilm eradication than either treatment alone [19,20], and the combination therapy was also recommended by Pirnay et al. (2024) as the most efficient treatment strategy [8]. For *P. aeruginosa*, the antibiotics that have been found to enhance the phage effect include ciprofloxacin, colistin, aztreonam, and gentamycin, and carbapenem antibiotics meropenem, ertapenem, and imipenem [8,19,20,21]. In this patient case, the two-week phage–meropenem treatment allowed the patient to be discharged after months of inpatient care and to return to her daily activities. No side effects related to the phage treatment during the phage therapy course or the 10-month follow-up period were observed. Now, like other patients of her age, she is occasionally experiencing infections arising from chronic underlying illnesses (ASO) and other common infections like pyelonephritis and COVID-19 (Table S1). All in all, we consider phage–meropenem combination therapy a success.

## 4. Materials and Methods

### 4.1. Human Subject

The patient was treated under article 37 of the Declaration of Helsinki [22]. The patient gave her written informed consent to undergo the bacteriophage treatment and for the case study to be published. Laboratory markers and infection values were monitored, with slight exceptions, as recommended by Khatami et al. [23] at Fimlab, Tampere (Table S1). In the PET-CT scan, 18F-FDG (fluorodeoxyglucose) uptake was used as the marker for tissue glucose metabolism, and a Siemens Biograph Vision PET-CT (Siemens, Erlangen, Germany) nuclear medicine scanner was used for imaging.

### 4.2. Phage Sensitivity Testing of the Patient Isolate

The *P. aeruginosa* patient isolate was named #7250. Lysogeny broth (LB) [24] was used as growth media, and LB supplemented with 1.5% and 0.4% was used as solid and soft-agar media, respectively. Phage culture and titration were performed as described earlier [24,25]. The preliminary phage sensitivity of *P. aeruginosa* #7250 was tested with a drop assay against 24 lytic *Pseudomonas* phages. Phages fNenPA2p2 and fNenPA2p4 (from the collection of PrecisionPhage Ltd., Jyväskylä, Finland) and fGstaPae02 (the collection of the University of Helsinki, Helsinki, Finland) were selected for the HFC-Pae10 cocktail. The potency of the three selected phages, individually and as a mixture, with regard to infecting *P. aeruginosa* #7250 in the presence of meropenem (200 µg/mL) was tested in liquid culture by monitoring a bacterial culture with oCelloScope (BioSense Solutions, Farum, Denmark) for 9 h 30 min using the method described earlier [25]. Each sample was analyzed in triplicate, and the calculation of mean and standard deviation, as well as the visualization of the results, was performed with OriginPro 2022b.

### 4.3. Phage Cocktail Production

Phages were produced in Vegitone LB (Merck Millipore, Darmstadt, Germany) solidified with 2% Agar No. 2 Bacteriological (Neogen, Lansing, MI, USA) using a method described elsewhere [15]. Phages were pre-purified by ultrafiltration with 100 kDa Vivaspin concentrators (Sartorius, Göttingen, Germany), after which, the phages were combined at a 1:1:1 ratio to form the HFC-Pae10 cocktail. The cocktail was purified with an EndoTrap HD column (LIONEX GmbH, Braunschweig, Germany) as described in [26] and stored in 0.9% NaCl + 8% sucrose. Endotoxin concentration was measured with an EndoLISA (bioMérieux, Marcy-l’Étoile, France) kit and a Hidex Sense microplate reader (Hidex, Turku, Finland). To prepare the final formulation administered to the patient, 750 µL of HFC-Pae10 was mixed with 500 mL of 0.9% saline at the hospital pharmacy.

### 4.4. Phage-Neutralizing Antibodies

To analyze if phage-neutralizing antibodies were formed during the phage treatment, serum samples were mixed 1:1 with the phage cocktail, the mixtures were incubated at RT for two hours, and the titers were compared to a non-treated phage cocktail.

## 5. Conclusions

In this work, phage–meropenem combination treatment was found to be an efficient and safe way to eradicate *P. aeruginosa* from artery stents and to prevent *P. aeruginosa*-induced septicemia. No adverse reactions were observed during the treatment.

## Data Availability

All data related to the treatment of the patient are included in the manuscript. The genomic sequence of phage fGstaPae02 is available on request from the corresponding author. For more information concerning phages fNenPA2p2 and fNenPA2p4, the reader is advised to contact PrecisionPhage Ltd.

## Supplementary Materials

The following supporting information can be downloaded at https://www.mdpi.com/article/10.3390/antibiotics13100916/s1: Table S1, Laboratory tests performed during phage therapy and the 10-month follow-up period.
